# Disparities in the use of remote general practice consultations: learning from the COVID-19 pandemic, an analysis of 19 million electronic health records using OpenSAFELY

**DOI:** 10.1186/s12916-025-04469-1

**Published:** 2025-12-02

**Authors:** Abodunrin Q. Aminu, David R. Sinclair, Katie Davies, Alex Hall, Milan Wiedemann, Terence W. O’Neill, Andrew Clegg, Chris Todd

**Affiliations:** 1 Policy Research Unit in Older People and Frailty/Healthy Ageing, School of Health Sciences, Faculty of Biology, Medicine and Health, National Institute for Health and Care Research (NIHR), The University of Manchester, Manchester, M13 9PL UK; 2 Manchester Academic Health Science Centre, Manchester, M13 9NQ UK; 3 National Institute for Health and Care Research (NIHR) Policy Research Unit in Older People and Frailty/Healthy Ageing, Population Health Sciences Institute, Newcastle University, Newcastle Upon Tyne, NE4 5PL UK; 4 Faculty of Medical Sciences, Population Health Sciences Institute, Newcastle University, Newcastle Upon Tyne, NE1 7RU UK; 5 School for Primary Care Research, Centre for Primary Care, Manchester Academic Health Science Centre, National Institute for Health and Care Research, University of Manchester, Manchester, M13 9PL UK; 6 Nuffield Department of Primary Care Health Sciences, Bennett Institute for Applied Data Science, University of Oxford, Oxford, UK; 7 Centre for Epidemiology Versus Arthritis, The University of Manchester, Manchester, M13 9PL UK; 8 NIHR Manchester Biomedical Research Centre, Manchester University Foundation NHS Trust, Manchester, M13 9WL UK; 9 University of Leeds & Bradford Teaching Hospitals NHS Foundation Trust, Leeds, LS2 9JT UK; 10 Manchester University NHS Foundation Trust, Manchester, M13 9WL UK

**Keywords:** Digitalisation, GP appointments, Health services, Older adults, Geriatrics, COVID-19, Health policy

## Abstract

**Background:**

Digital technologies are crucial to drive the needed improvement in NHS primary care delivery and access. The impact of these digital interventions on health inequalities remains a critical area of concern and uncertainty. Transition to digital primary care services was rapidly accelerated during the COVID-19 pandemic. We explored what can be learnt from this transition to digital access by examining the patterns of remote general practice consultation before and after the pandemic and the influence of age, gender, social deprivation, and ethnicity on these patterns.

**Methods:**

This is a longitudinal study in primary care settings involving data from19 million men and women aged 18 + years registered with general practices in England between January 2019 and February 2022 using the OpenSAFELY platform. The main outcome was remote consultation (telephone, video, or electronic) of all appointments recorded by GPs. Binomial regression models including marginal effect probabilities were used to analyse the proportion of remote consultations in all appointments. Covariates including age, gender, deprivation, and ethnicity were adjusted for in the models.

**Results:**

Remote consultations increased from 10.1 million per annum (March 2019 to March 2020) to 32.7 million per annum during the pandemic (March 2020 to March 2022). Pre-pandemic, 85 + year olds had the highest probability of remote consultation (0.133, 95% CI [0.132–0.133]). In the pandemic period, the probability of remote consultation increased for all age groups with those aged 18–49 years having the highest probability of remote consultation and those aged 85 + having the second highest probability. Men were less likely to have remote consultations than women pre-pandemic and in the 2 years after the pandemic started. Before the pandemic, the most affluent group (5th deprivation quintiles) had the lowest probability of having consultation being held remotely (0.086, 95% CI [0.086–0.086]), a trend that was maintained through the first 2 years of the pandemic. White ethnic group had the highest probabilities of remote consultations across the study period.

**Conclusions:**

There were significant variations in remote consultations by age, gender, socioeconomic group, and ethnicity during the pandemic. These factors should be considered when planning access to services especially for vulnerable patients.

**Supplementary Information:**

The online version contains supplementary material available at 10.1186/s12916-025-04469-1.

## Background

The COVID-19 pandemic precipitated considerable changes in the delivery of UK primary healthcare with an accelerated shift towards digital technologies including telephone, video, and electronic communication to support the delivery of health care [1]. The use of digital technologies to access and deliver healthcare services is not new. The World Health Organization (WHO) has long advocated for the integration of digital technologies to enhance healthcare delivery, acknowledging their expanding role in modern society [2]. Prior to the pandemic, National Health Service (NHS) England had already begun prioritising the digital transformation of primary care through initiatives such as the Long Term Plan [2], which set out ambitions for a ‘digital-first’ primary care model. This was reinforced by the Digital First Primary Care programme, aimed at enabling patients to access care remotely via online consultations and triage tools. The pandemic accelerated this agenda, prompting widespread adoption of remote technologies out of necessity. In response, NHS England introduced the Modern General Practice Access framework, which formalised the use of digital platforms for appointment booking, clinical triage, and consultation delivery [3]. NHS England’s post-pandemic plan for recovering access to primary care outlines the implementation of ‘Modern General Practice Access’ via online requests and digital telephony to tackle the well-known problem of the ‘8am rush’ of patients having to telephone their GP first thing in the morning to make an appointment [4]. This strategic alignment is reinforced by the broader agenda for a national transformation of digital access to public services [5]. However, the NHS remains confronted with the challenges of patients’ inadequate access to general practitioners (GP), a shortage of GPs in deprived areas leading to regional variations, underinvestment in community funding, and an urgent need for primary care to benefit from digital systems, as identified in the 2024 Darzi report [6].

Expanding access to equitable and inclusive remote primary care is a global health priority [7], as digital consultations offer scalable solutions to improve healthcare access, efficiency, and resilience across diverse health systems. Remote GP consultations offer several advantages that have made them a vital component of modern primary care, particularly during the COVID-19 pandemic [8]. They provide greater convenience and flexibility for patients, reducing the need for travel, time off work, or childcare arrangements—factors that can otherwise pose barriers to accessing care. For practices, remote consultations can improve workflow efficiency by enabling better triage and allocation of clinical time [9]. They also help maintain continuity of care during public health emergencies by minimising infection risk in clinical settings. Remote consultations have been shown to be effective for managing mental health issues, alcohol misuse, weight management, and smoking cessation [10]. Despite the opportunities with remote GP services, the increased transition to digitalised health services raises concerns about potentially amplifying health inequalities from the inadvertent digital exclusion of certain demographic groups [11]. A previous study found that remote consultations, particularly those conducted via the phone or emails, may suffer from reduced communication quality [12] as well as inability to perform clinical assessments and removal of clinically important information (e.g., olfactory cues) when compared with face-to-face consultations. This can be challenging for patients with cognitive or communication difficulties and underscores the concerns about the digital and social exclusion, especially for older adults during the pandemic [13–16]. Digitalised health services have also raised patient safety concerns, albeit rarely [17]. Technical failures, such as technology infrastructure breakdowns and software malfunctions, can disrupt the delivery of care and compromise patient safety. Information-related issues, including incorrect display of patient data and missing documentation, further exacerbate these risks by potentially leading to misdiagnoses or inappropriate treatment plans [18]. Human–computer interaction challenges also arise, as healthcare providers may struggle with inadequate training or the limitations of online consultation tools, impacting clinical assessments.

Although several United Kingdom (UK) based studies have explored aspects of this transition to digital or online services in primary care during the pandemic, most have relied on survey data, small clinical samples, or single-provider datasets, limiting generalisability. For example, Murphy et al. [19] conducted a mixed-methods evaluation of remote consultation implementation from the perspective of practice staff, while Turner et al. [20] and Verity and Brown [21] provide qualitative insights into the unintended consequences and challenges of remote access for vulnerable populations. Although these studies offer valuable context, they primarily reflect practitioner or small-group experiences and do not quantify trends across the broader population. Other studies investigated specific facets of digital service delivery in the UK, such as mental health support via remote consultations [22], patient perceptions of virtual care [23], and digital facilitation initiatives in general practice [24]. Darley et al. [25] assessed how online consultation systems affect care quality, but their findings focused largely on system design rather than on patient demographics or usage disparities. Collectively, these studies have contributed important insights, yet few have examined population-wide, longitudinal patterns of remote primary care access stratified by age, gender, ethnicity, and deprivation. There is a lack of robust evidence based on large-scale electronic health record data that tracks digital uptake over multiple years and evaluates whether disparities in access have emerged or persisted. Our study addresses this gap by analysing a large database of electronic health records across a three-year period, offering one of the most comprehensive assessments to date of digital primary care access and equity in England during the pandemic.

The shift to use of digital technology in delivery of care during the pandemic provided an opportunity to explore how the method of access to services varied in different population groups including older people [19] and enables learning from this transition to digital access during the pandemic. Using data from the OpenSAFELY (https://www.OpenSAFELY.org/) platform, the aims of our analysis are to examine (i) how remote consultations varied by age group before (March 2019–March 2020) and after the onset of the first national lockdown in the UK (March 2020–March 2022) and (ii) the impact of gender, ethnicity, and socioeconomic status on the proportion of use of remote consultation.

## Methods

### Study design

A retrospective longitudinal study using pseudo-anonymised patient data from healthcare consultation records in England using the OpenSAFELY platform. OpenSAFELY is a secure research environment for accessing electronic health records (EHR) created during the COVID-19 pandemic to facilitate urgent research. This design enabled us to evaluate trends over time in the use of remote general practice consultations and to examine associations with demographic and socioeconomic.

### Settings

General practices across England used The Phoenix Partnership (TPP) SystmOne software. OpenSAFELY procures access to the TPP data, which allows for the analysis of electronic health records of 24.2 million individuals presently registered across 2546 general practices and covering approximately 43% of all practices in England [26]. Prior validation research have demonstrated that TPP data is broadly representative of the English population, with data completeness comparable to census proportions across England [27]. Nonetheless, TPP coverage as a proportion of the ONS population varies across regions, with the highest representation in the East of England (91%) and East Midlands (86%), and the lowest in London (19%), South-East England (18%), and West Midlands (17%) [27]. Although the geographic coverage of TPP varies across regions, key demographic measures—including age, sex, ethnicity, and deprivation—within the TPP population are closely aligned with the Office for National Statistics benchmarks, with discrepancies generally within one percentage point [27]. This alignment supports the representativeness of the TPP data to the English population.

### Observation periods

Based on the anniversary of the announcement of the first UK-wide national lockdown on 23rd March 2020 during the COVID-19 pandemic, the study was divided into three periods:

- Pre-pandemic year: 23rd March 2019–22nd March 2020
- First pandemic year: 23rd March 2020–22nd March 2021
- Second pandemic year: 23rd March 2021–22nd March 2022

### Study population: inclusion and exclusion criteria

Patients were included in the analysis for a given year if they had an active registration with a TPP general practice on the first day of the corresponding 12-month observation period. Patients could contribute to multiple annual cohorts if they remained continuously registered over time.

Inclusion criteria:

- Adults aged ≥ 18 years
- Registered with a TPP general practice in England
- Registration for at least one of the three study periods (2019–2020, 2020–2021, or 2021–2022)
- At least one recorded consultation event during the relevant period

Exclusion criteria:

- Patients with no registration record on the start date of any the observation periods
- Patients with no recorded consultations

These criteria were designed to maximise data quality and ensure that included patients had adequate exposure time within each observation window. Excluding those without any consultation ensured that results reflected actual service utilisation and not unobserved denominators.

### Variables

#### Outcomes

Primary outcome: The proportion of remote appointments was the primary outcome in this study. Remote consultations were identified by querying Systematised Nomenclature of Medicine (SNOMED) codes that correspond to remote consultation events (telephone, computer link, video, text, remote, e-consultation) recorded by GP. Additionally, we used appointment records to investigate total consultations (denominator). There is no direct, explicit relationship between an appointment and a consultation; but if an appointment is recorded then a corresponding consultation will be created. Combined with the remote consultations data (numerator), this systematic approach ensures transparent and reproducible outcome measurement for calculating annual proportions [26].

#### Covariates

1. Age was categorised into five age bands: 18-to-49 years, 50-to-64 years, 65-to-74 years, 75-to-84 years, and 85 + years.
2. Gender was categorised into male and female.
3. Ethnicity was grouped into the Office for National Statistics’ higher-level categories and contracted to ‘Asian or Asian British’, ‘Black or Black British’, ‘mixed’, ‘White’, and ‘other ethnic group’ for individuals who do not identify with any of the other four categories [28].
4. Deprivation status was based on scores from the English Index of Multiple Deprivation (IMD) [29]. IMD is an area-based composite score assigned to each patient based on their residential postcode at the Lower Super Output Area (LSOA) level. The IMD is derived from seven domains including income, employment, health, education, crime, housing, and environment. In OpenSAFELY, each patient’s IMD is linked to their primary care record via the TPP electronic health system and categorised into national quintiles (1 = most deprived; 5 = least deprived) based on the 2019 English national rankings. IMD was treated as a categorical variable in all analyses to capture socioeconomic variation in remote consultation use.

The covariates included in our analysis—age, gender, ethnicity, and area-level deprivation—were selected based on evidence demonstrating their significant association with both healthcare utilisation and digital access. Age is a key determinant of consultation behaviour, with younger and older adults exhibiting different preferences and needs for remote care, as shown in previous UK studies [19, 23]. Gender has consistently been associated with healthcare-seeking behaviour, with women more likely to engage with primary care services than men [30]. Ethnicity has been linked to differences in health access, communication preferences, and digital literacy, with minority ethnic groups often facing structural barriers in using remote health services [31]. Socioeconomic status, based on IMD, is a known predictor of both health outcomes and digital exclusion, with people in more deprived areas less likely to have stable internet access or digital literacy (Office for National Statistics, 2020). Including these variables was essential to evaluate potential disparities in access to remote consultations during the pandemic.

### Data source

All data were analysed via the secure OpenSAFELY environment. Data were pseudonymised, encrypted, and analysed under NHS England information governance protocols (see supplementary material). Researchers had no direct access to the TPP electronic health records and only developed analytic scripts using dummy data, which are to run against an OpenSAFELY backend servers hosting the real data.

### Statistical analysis

Descriptive statistics including counts, percentages, and proportions were used to describe the monthly frequency of remote consultations and appointments over the study period. We also examined the proportion of remote consultations, dividing total remote consultations recorded in each 12-monthly study periods by the total number of appointments in the same period. To contextualise the results in terms of population size, the total number of consultations made during the study period was compared alongside with the size of the England adult population census data by age group.

We conducted binomial regression with marginal effects to quantify variations in the probability of remote consultations by age groups. This modelling approach was selected for its interpretability and suitability for binary outcome data. Unlike logistic regression, which provides odds ratios that can be challenging to interpret for non-specialist audiences—particularly when outcomes are common—binomial regression with marginal effects yields predicted probabilities that are easier to communicate and compare across groups. This approach aligns with public health practice and enhances accessibility of the results to clinicians and policymakers, while our model adjusted for known confounders available within the OpenSAFELY platform, such as demographics and deprivation. In this analysis, the proportion of remote consultations were derived by dividing remote consultations by the total appointments for each 12-monthly study period. We conducted a complete case analysis, including only individuals with non-missing data for the relevant variables in each model. After fitting the binomial models, we calculated the marginal (effect) probabilities using average adjusted predictions [32]. Adjusting for gender, ethnicity, and socioeconomic deprivation (IMD) in the binomial regression model allows us to estimate the independent association of each factor with the probability of using remote consultations, while controlling for potential confounding from other variables in the model. The interactions of time-period with age, gender, ethnicity, and area deprivation were evaluated. We also included interaction terms between age and gender and other covariates in our binomial regression models and present the results to explore compounding effects (see supplementary material for details). The models were implemented using R 4.3.1 with the ‘marginaleffects’ and ‘stats’ packages. Outputs are reported as odds ratios (ORs) and probability (PR) with 95% confidence intervals (CIs).

As a form of sensitivity check, prior to the main analysis, we investigated possible ambiguous appointments by generating appointment records using six different statuses individually and altogether (‘arrived’, ‘in progress’, ‘finished’, ‘visit’, ‘waiting’, ‘patient walked out’). We found that the appointment status ‘arrived’ was consistently used by practitioners and generated the most outcome.

## Results

### Changes in remote consultations and all appointments by age group and other demographic variables in the pre-pandemic and pandemic periods

Tables 1 and 2 (including Additional file 1: Figs. S1–S4) show the numbers of remote consultations and total appointments for each of the 3 years in the study period. Remote consultations accounted for 23.9% of the overall appointments in the study period (Table 1). Remote consultations increased from 10.1 million in the pre-pandemic year (March 2019–March 2020) to 32.7 million in the first pandemic year (March 2020–March 2021), representing an increase of 22.6 million consultations, or approximately 224% (Table 2). Initially, there was a drop in the total number of appointments in the first year of the pandemic, nonetheless the total number increased by 16% between March 2021 and February 2022, compared to the pre-pandemic period.

**Table 1:** Combined numbers of remote and total primary care appointments by patients in OpenSAFELY, from 23 March 2019 to 22 March 2022. Numbers rounded to the nearest thousand

Group	Remote (1000 s)	Appointments (1000 s)	Percentage remote (%)
Total	73,881	309,403	23.9
Age			
18–49	30,564	115,407	26.5
50–64	17,456	75,741	23.0
65–74	10,977	53,598	20.5
75–84	9560	44,368	21.5
≥ 85	5324	20,289	26.2
Gender			
Women	46,709	188,101	24.8
Men	27,172	121,302	22.4
Ethnicity			
Asian or Asian British	4052	18,742	21.6
Black or Black British	1316	5685	23.1
Mixed	695	2810	24.7
Other ethnic groups	727	3184	22.8
White	50,568	212,897	23.8
(Missing ethnicity)	(16,523)	(66,085)	(25.0)
Deprivation quintile			
Most deprived	14,435	58,947	24.5
2nd	14,426	58,625	24.6
3rd	15,485	64,021	24.2
4th	14,438	61,275	23.6
Least deprived	12,715	57,083	22.3
(Missing deprivation)	(2382)	(9452)	(25.2)

**Table 2:** Number of remote and total primary care appointments by patients in OpenSAFELY, from 23 March 2019 to 22 March 2022. Numbers rounded to the nearest thousand

Group	Pre-pandemic period			Pandemic period					
	Mar 2019–Mar 2020			Mar 2020–Mar 2021			Mar 2021–Mar 2022		
	Remote consultations (1000 s)	Appointments (1000 s)	Percentage remote (%)	Remote consultations (1000 s)	Appointments (1000 s)	Percentage remote (%)	Remote consultations (1000 s)	Appointments (1000 s)	Percentage remote (%)
Total	10,031	98,740	10.2	32,712	96,014	34.1	31,139	114,649	27.2
Age									
18–49	3908	36,984	10.6	13,516	35,714	37.8	13,140	42,709	30.8
50–64	2261	23,851	9.5	7724	23,403	33.0	7471	28,487	26.2
65–74	1541	17,685	8.7	4891	16,412	29.8	4545	19,501	23.3
75–84	1426	14,118	10.1	4226	13,836	30.5	3908	16,414	23.8
≥ 85	895	6102	14.7	2355	6649	35.4	2074	7538	27.5
Gender									
Women	6316	59,637	10.6	20,658	58,710	35.2	19,735	69,754	28.3
Men	3715	39,103	9.5	12,054	37,304	32.3	11,403	44,896	25.4
Ethnicity									
Asian or Asian British	535	5810	9.2	1771	5688	31.1	1745	7243	24.1
Black or Black British	171	1751	9.8	580	1765	32.9	565	2170	26.0
Mixed	86	844	10.1	303	877	34.5	307	1090	28.2
Other ethnic groups	86	981	8.8	308	940	32.7	333	1264	26.4
White	6580	66,326	9.9	22,164	65,834	33.7	21,823	80,737	27.0
(Ethnicity missing)	2573	23,029	11.2	7586	20,910	36.3	6365	22,146	28.7
Deprivation quintile									
Most deprived	1973	18,678	10.6	6380	18,387	34.7	6082	21,881	27.8
2nd	1987	18,626	10.7	6390	18,241	35.0	6048	21,758	27.8
3rd	2133	20,451	10.4	6856	19,857	34.5	6495	23,713	27.4
4th	1957	19,669	9.9	6383	18,943	33.7	6098	22,663	26.9
Least deprived	1680	18,427	9.1	5665	17,648	32.1	5370	21,008	25.6
(Deprivation missing)	301	2889	10.4	1037	2937	35.3	1044	3626	28.8

Overall, the proportion of remote consultations relative to total primary care appointments varied throughout the study. Specifically, the proportion of remote consultations rose from 10.2% (10,031/98,740) before the pandemic to 34.1% (32,712/96,014) and 27.2% (31,139/114,649) in the first and second years of the pandemic respectively (Table 2). In terms of stratification by population size, data from England 2021 census shows that while 53.7% (24,533,915/45,691,633) of the adult (18 +) population is aged 18–49 this age group made 37.3% (115,407/309,402) of the consultations identified in our study. Conversely 85 + year olds comprise 3.2% (1,454,740/45,691,633) of the adult population but had 6.6% (20,289/309,402) of the consultations, and 75–84 year olds make up 4.7% (2,171,788/45,691,633) of the population but made 14.3% (44,368/309,402) of the consultations (Additional file 1: Table S1).

### Probability of remote consultation by age group

**Fig. 1:**
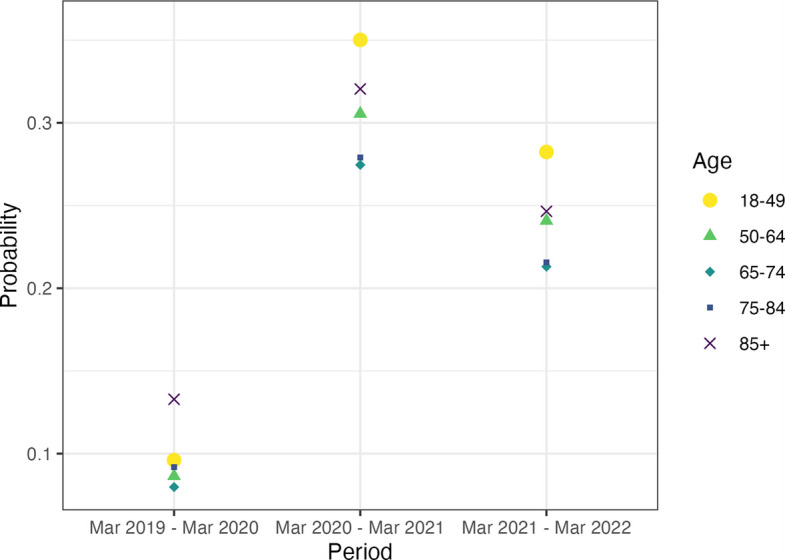
Probability that a consultation was held remotely by age group and time-period (23 March 2019 to 22 March 2022). 95% confidence intervals are not plotted due to their small sizes (min range: 0.0004, max range: 0.0011); CIs are included in Additional file 1: Table S2

We determined the probability that a consultation was held remotely for each age group, gender, ethnicity, and deprivation quintile. Details of the odds ratio from the regression model and the marginal effect probabilities can be found in the supplementary material (Additional file 1: Tables S2–S6). The probability (pr) that a person aged 85 + had their consultation held remotely was pr = 0.133 [95% CI 0.132 to 0.133], the highest of any adult age group in the pre-pandemic period analysed compared to those aged 18–49 years old (Fig. 1).

In the first year of the pandemic (March 2020–March 2021), 18–49 years were most likely to have a consultation held remotely pr = 0.350 (95% CI 0.350 to 0.351), with people aged 85 + the second most likely pr = 0.321 (95% CI 0.320 to 0.321). A U-shaped association with age emerged, with the youngest and oldest groups more likely to have a consultation held remotely than 50–64-year-olds, 65–74-year-olds, and 75–84-year-olds in the first pandemic year. This association holds in the second pandemic year (March 2021 to March 2022), but the probabilities drop for all age groups. As shown in Additional file 1: Table S2, we found 85 + year olds had 11-point difference in the probability of a consultation being held remotely from March 2019 (pr = 0.133) to March 2022 (pr = 0.247), which is the lowest compared to the 19 points rise among the 18–49 year olds (pr = 0.096 to pr = 0.282), the 15 points rise among the 50–64 year olds (pr = 0.086 to pr = 0.241), the 13 points rise among 65–74 years (pr = 0.08 to pr = 0.213), and the 12 points rise among the 75–84 years (pr = 0.092 to pr = 0.216).

### Probability of remote consultation by gender

**Fig. 2:**
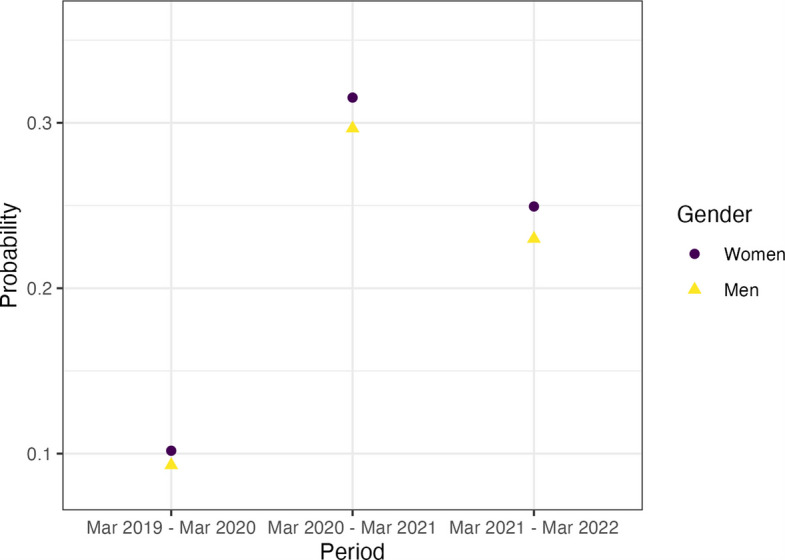
Probability that a consultation was held remotely by gender and time-period (23 March 2019 to 22 March 2022). 95% confidence intervals are not plotted due to their small sizes (min range: 0.0004, max range: 0.0007); CIs are included in Additional file 1: Table S3

Women were consistently more likely than men to have consultations held remotely. The probability of women having remote consultations in the period March 2019 to March 2020 was pr = 0.102 (95% CI 0.102 to 0.102) compared to pr = 0.0930 (95% CI 0.093 to 0.093) for men. This pattern was sustained in the pandemic years as women had a probability of remote consultations pr = 0.315 (95% CI 0.315 to 0.316) and pr = 0.249 (95% CI 0.249 to 0.250) compared to men pr = 0.297 (95% CI 0.296 to 0.297) and pr = 0.230 (95% CI 0.230 to 0.230) in periods March 2020 to March 2021 and March 2021 to 2022 respectively. This indicates the relative differences between women and men are very similar throughout the 3 years analysed (Fig. 2).

### Probability of remote consultation by deprivation (IMD)

**Fig. 3:**
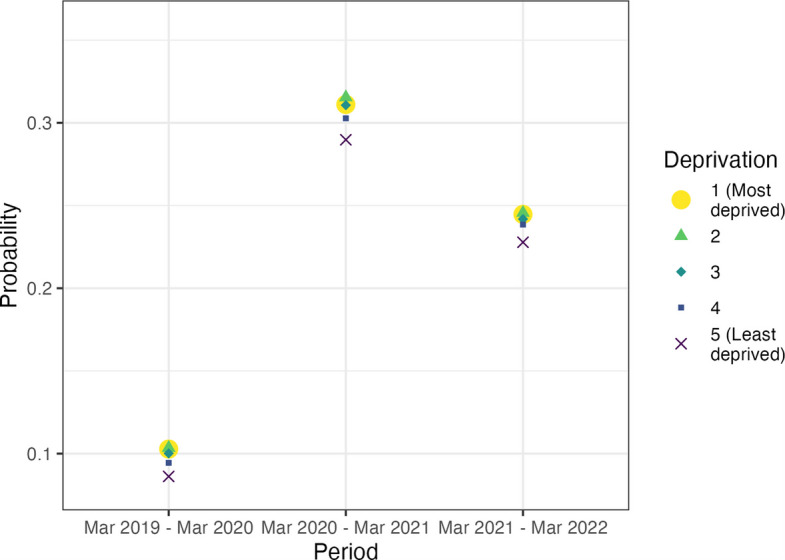
Probability that a consultation was held remotely by deprivation group and time-period (23 March 2019 to 22 March 2022). 95% confidence intervals are not plotted due to their small sizes (min range: 0.0005, max range: 0.0008); CIs are included in Additional file 1: Table S4

People living in the affluent areas (1st and 2nd deprivation quintiles) were the least likely to have a consultation held remotely in all three periods compared to those in the most deprived areas. Before the pandemic, the probability of the most deprived people having a consultation held remotely was pr = 0.103 (95% CI 0.103 to 0.103) compared to pr = 0.086 (95% CI 0.086 to 0.086) for the least deprived. In the first year of the pandemic, there was an overlap in the probability of remote consultation for those in 1 st and 3rd deprivation quintiles, and those in the 2nd deprivation quintile had 31.5% probability of remote consultations pr = 0.315 (95% CI 0.315 to 0.316) while the least deprived quintile had 29% probability pr = 0.290 (95% CI 0.290 to 0.290) of having consultation being held remotely. Similar differences in the probability of remote consultations between the deprivation quintiles continued in the second year of the pandemic (Fig. 3).

### Probability of remote consultation by ethnicity

**Fig. 4:**
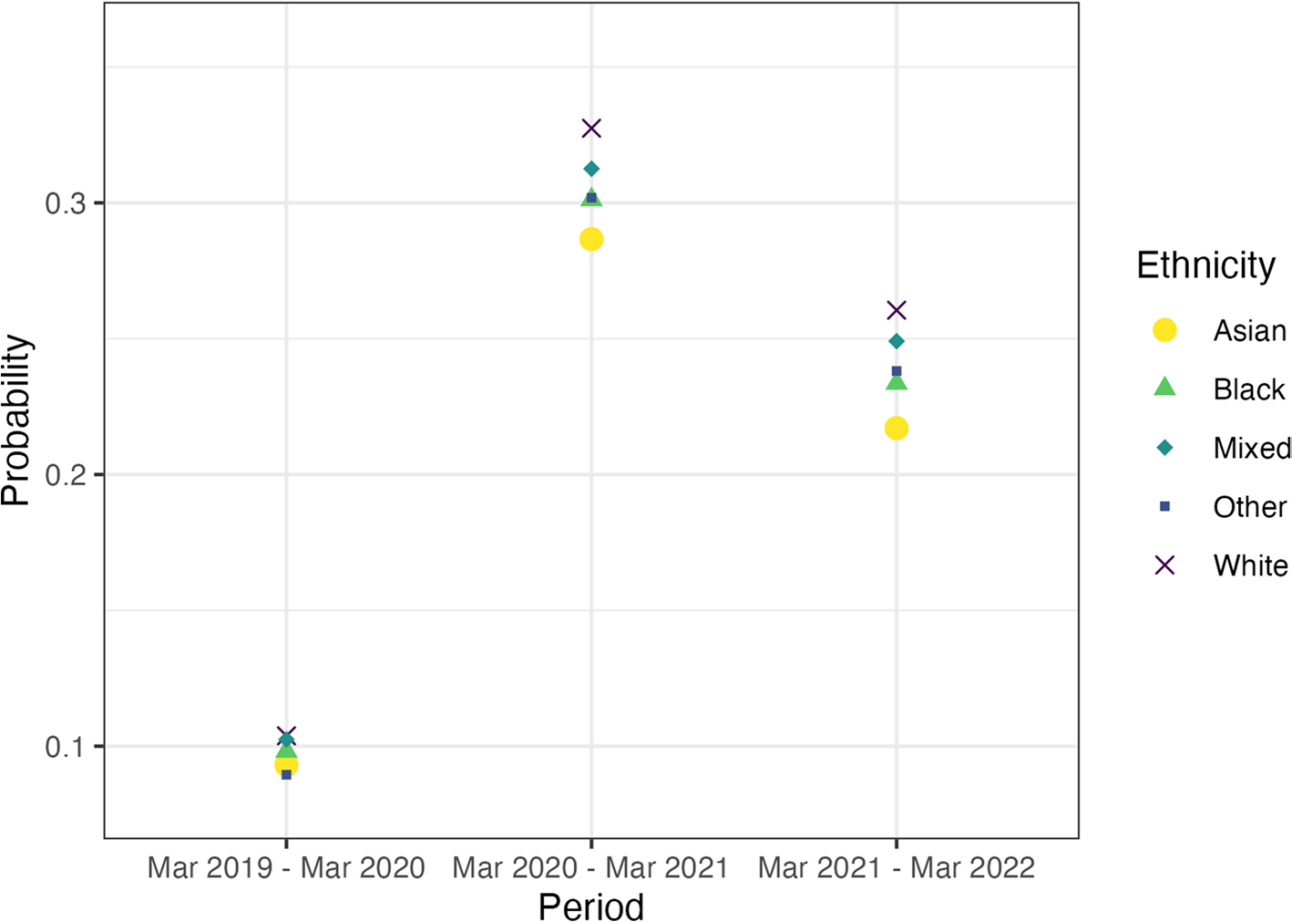
Probability that a consultation was held remotely by ethnicity and time-period (23 March 2019 to 22 March 2022). 95% confidence intervals are not plotted due to their small sizes (min range: 0.0002, max range: 0.002); CIs are included in Additional file 1: Table S5

Figure 4 presents the probability of remote consultations for different ethnicities. In each of the 3 years analysed, White people were the ethnic group most likely to have a consultation held remotely. In both years of the pandemic, people who are Asian, Black, or have a mixed ethnic background had a smaller relative increase in the probability of a remote consultation than people who are from White ethnicity. For instance, people who are White had 22 points increase in remote consultations from pre-pandemic period (pr = 0.104) to first pandemic period (pr = 0.328) than people who are Asian with a 19 points rise (pr = 0.093 to pr = 0.287); people who are Black with a 20 points rise (pr = 0.098 to pr = 0.301); people from other ethnic backgrounds with a 21 points rise (pr = 0.090 to pr = 0.302); and people with a mixed background with 21 points rise (pr = 0.103 to pr = 0.313).

### Interaction effect between the three observation periods and the covariates (age, gender, ethnicity, and deprivation)

Interaction effects by period showed that demographic associations with remote consultation use shifted across the three-time frames (Additional file 1: Table S6). Pre-pandemic, patients aged 85 + had significantly higher odds of remote consultations compared to those aged 18–49 (OR 1.44; 95% CI: 1.43–1.44), but this reversed during the pandemic, with notably lower odds in 2020–2021 (OR 0.60; 95% CI: 0.60–0.61) and 2021–2022 (OR 0.57; 95% CI: 0.57–0.58) among the 85 + years compared to those in the 18–49 years group. A reversal was also observed in the deprivation gradient. In the pre-pandemic year, those in the least deprived quintile had lower odds of remote consultations than the most deprived (OR 0.82; 95% CI: 0.82–0.83), whereas by 2021–2022 those in the least deprived quintile had higher odds (OR 1.10; 95% CI: 1.10–1.11) compared to the most deprived group. Ethnic differences shifted subtly: patients from ‘other ethnic’ group had lower odds pre-pandemic (OR 0.84; 95% CI: 0.84–0.85) but higher odds during both pandemic years (OR 1.04; 95% CI: 1.03–1.05) compared to those in the White ethnic group. ‘Asian’ and ‘Black’ patients saw modest increases in relative odds during the pandemic but remained less likely to use remote consultations compared to ‘White’ patients.

## Discussion

In this study, we investigated the patterns and variations in remote consultations prior to and during the COVID-19 pandemic. Those aged 85 + year had the largest proportion of remote consultations pre-pandemic, but younger adults were more likely to have remote consultations during the first 2 years of the pandemic. The interaction effects with time further highlight the reversal of consultation odds among the oldest age group, which could likely reflect shifts in service design, prioritisation, or digital readiness during the pandemic. There was a gender-gap in remote consultations, and differences by ethnicity and deprivation status. The probability of consultation being held remotely was highest among women (compared to men), those from the three more deprived quintiles 1 st, 2nd, and 3rd (compared to the most affluent people in the 4th and 5th quintile) and those from White ethnicity compared to others.

The adoption of remote consultations in primary care has presented both opportunities and challenges. We found an increase in total appointments during the pandemic period compared to the pre-pandemic period. NHS England have reported a similar trend, with recent data showing an extra 56.3 million appointments (363.6 million excluding COVID vaccinations) or 63.4 million (370.7 million including COVID vaccinations) over the last year (2023/2024) compared to 2018/2019 [33]. This has been attributed to the delivery plan for recovering access to primary care, which included implementing digital access to GP practices. Other studies have also reported an increase in the number of remote appointments during the pandemic [19, 34]. Digitalised services in primary care may improve efficiency, with reduced costs and time savings for healthcare providers [8], and can help to improve access more generally as shown in the present study. However, patients seen remotely have reported feeling less supported in making informed choices autonomously in virtual settings [8]. There have also been reports of increased patients’ satisfaction in practices that provide higher face-to-face appointments compared to virtual, especially among those aged 65 years and over [35]. It is therefore important for proper clinical assessment in determining which patient groups will benefit from virtual settings to avoid less effective care.

Due to digital barriers, consultations are often made by proxies for oldest old people living either in their own homes/community or for those in residential care [36]. This will possibly explain why the largest proportion of remote consultations was seen among those aged 85 years and over prior to the pandemic. Overall, our findings suggest that digitalisation of primary care services during the pandemic have favoured the younger age groups more in terms of facilitating remote consultations. This trend raises concerns about potential digital and social exclusion of older adults. The higher uptake of remote consultations among younger adults may be partly explained by the nature of health concerns more commonly managed in this age group, such as mental health conditions, alcohol and tobacco use, and weight management—many of which are well-suited to remote consultation formats. Previous research has shown that younger adults are more likely to seek care for anxiety, depression, and lifestyle-related concerns, particularly during the pandemic [37]. These conditions often do not require physical examination and can be effectively addressed through telephone or video consultations, potentially contributing to the greater uptake observed in this group. While most remote consultations are conducted via telephone, which may be accessible for older adults, this demographic could still encounter challenges in accessing appointments especially with the transition to digital Voice over Internet Protocol (VoIP) [5]. Many healthcare practices utilise remote communication methods, such as text messaging for information gathering and questionnaires (e.g., related to medications or chronic disease reviews) [38]. This reliance on digital tools underscores the importance of addressing the digital divide. Women had a higher proportion of remote consultations compared to men. Men are less likely to seek primary care in the first place, so perhaps tend to seek care when things are more severe and thus more likely to benefit from face-to-face assessment [30]. Our findings on gender differences in remote consultation align with existing research indicating gender-based disparities in healthcare-seeking behaviour, with women generally being more proactive in seeking medical advice and accessing healthcare services [39].

Socioeconomic inequality is a risk factor for healthcare access [40, 41]. We found that people from more deprived socioeconomic areas had higher probabilities of consultation being held remotely compared to those from affluent areas. There are fewer GPs per person in deprived areas in England, a case of inverse care law where people who need the most healthcare are least likely to receive it [42, 43]. This is consistent with the Darzi report showing a shortage of GPs in deprived areas and underinvestment in community funding [2]. People from socioeconomically disadvantaged backgrounds often encounter numerous barriers when trying to access healthcare services. These barriers include financial constraints, difficulties with transportation, and a lack of healthcare facilities and staff in their local areas. While social deprivation plays a significant role in determining access to primary care services, there has been little research comparing the probability of remote consultations across different levels of deprivation, as our study has done. It is important to note that remote consultations may not be suitable for all appointment types, particularly for those with complex conditions. Especially for those where in-person doctor-patient communication is/was considered beneficial or where in-person investigations are required [44]. Given that social deprivation is often associated with more serious health issues [45], it is crucial to conduct further research. Specifically, there will be need for further studies to examine how remote consultations affect different health outcomes. This research would help ensure that the shift towards remote healthcare does not inadvertently widen existing health disparities.

The expansion of remote general practice consultations during the COVID-19 pandemic provides lessons for health systems worldwide. A comparative analysis by Fisk et al. [46] showed that while the UK’s centralised NHS enabled a rapid and coordinated shift to telehealth, other countries like the USA and Australia experienced more fragmented or policy-dependent uptake due to decentralised structures or temporary reimbursement incentives. Despite these structural differences, all systems may face common challenges around digital equity, access, and sustainability. Nationally, the findings from our study are relevant now that accessibility to primary care services is at the heart of NHS England 10years ‘fit for future’ policy [47] and can be helpful to stimulate recommendations for the needed NHS reforms [6]. There may be a need to re-examine the comprehensiveness of policies for improving access to ensure digital inclusion and address socioeconomic disparities while maintaining high-quality care. Key initiatives should include targeted digital support programmes for older adults, not necessarily to maintain their pre-pandemic levels of remote consultation usage, but to close the gap between all age groups while maintaining higher levels of remote appointments than the pre-pandemic level. Alongside, there is a need for gender-specific outreach strategies to encourage men’s engagement with remote healthcare services. Socioeconomic disparities can be addressed by encouraging investments in digital infrastructure and support in deprived areas, complemented by an integrated care model that considers social determinants of health [6, 48]. Following the Darzi report, these efforts can be underpinned by a reallocation of NHS resources to increase investment in community and primary care services, including significant capital investment in technology infrastructure.

There are caveats or limitations to take into consideration from our study. Firstly, we assume that each primary care provider codes remote consultations in a consistent manner. The data does not track patients between practices and as such may not include information for patients who have not had a continuous registration with their practice during the period analysed. Secondly, there were no adequate metadata available from OpenSAFELY to discern how appointments have been counted. In this study, we assume that a patient can only have one status (i.e., arrived, finished) per appointment, and if a patient has more than one of these, we only count a single appointment. We did not distinguish urban and rural settings, and practice-level characteristics were not accessible at the time of analysis. As a result, we could not account for geographic or contextual variation in digital consultation uptake—an important area for future research using regional or practice-level identifiers. Information on the clinical reasons, comorbidities, or types of diagnoses associated with each consultation would have been helpful to assess whether patterns in remote consultation use reflect differences in care needs, urgency, or suitability for remote delivery. This is an area that can be considered for future research to explore how different groups of patients benefit or otherwise are affected by the digitalisation of primary care services. Patients may be clustered within general practices, creating a potential hierarchical structure in the data that was not accounted for in this study. As consultation practices may vary between practices, this could potentially influence the observed associations. Another limitation of this study is that we used IMD as a measure of socioeconomic status (SES) rather than individual measures of SES. Thus, our results may be subject to the ecological fallacy. However, primary care records do not necessarily collect individual SES data reliably which would have introduced another source of uncertainty. Also, we could not reliably disentangle the telephony appointments from other remote appointment types as some of the SNOMED codes are ambiguous (i.e., we cannot check the difference between a telephone call with VoIP using a mobile phone and an audio-only call with VoIP using a tablet).

## Conclusions

Increased use of digital services is part of NHS England policy for improving access to primary care and aligns with policy for a national transition to digital telephony scheduled for completion in 2025. The shift in digitalisation from the pandemic enabled us to analyse disparities in remote GP consulting for different demographic and socioeconomic groups. We found that 85 + year olds had the largest proportion of remote consultations pre-pandemic, but younger adults were more likely to have remote consultations during the first 2 years of the pandemic. There is also a persistent gender-gap in remote consultations, and differences by ethnicity and deprivation status. While digitalisation offers an opportunity to reshape the healthcare landscape, it could exacerbate inequality. Therefore, this research highlights potential concern around the implications of the differences in remote consultations in terms of healthcare inequalities. Key policy initiatives focusing on addressing these differences will be instrumental in reforming the NHS primary care service delivery when creating access to service models. Further research is required to understand causative factors and outcomes in primary care digital services and to devise safe primary care provision models.

## Supplementary Information

Additional file 1: Figures S1–S4. Fig. S1 Monthly proportion of remote consultations by age group. Fig. S2 Monthly proportion of remote consultations by gender. Fig. S3 Monthly proportion of remote consultations by ethnicity. Fig. S4 Monthly proportion of remote consultations by area deprivation quintiles. Area deprivation measured by Index of Multiple Deprivation. Tables S1–S6. Table S1 Total consultations March 2019 to March 2022 by age group and population size by age group. Table S2 Probability of a consultation being remote by age group and period. Table S3 Probability of a consultation being remote by gender and period. Table S4 Probability of a consultation being remote by area deprivation quintile and period. Table S5 Probability of a consultation being remote by ethnic group and period. Table S6 Binomial regression of the proportion of remote consultations including interaction with period

## Data Availability

Data cannot be shared publicly due to governance restrictions. Access to the underlying raw pseudonymised patient data is restricted to approved researchers on the OpenSAFELY platform. All code for data management, analyses, and codelists used in this study is freely available at [GitHub - opensafely/digital-access-to-primary-care: Digital access to primary care for older people during COVID] (https://github.com/opensafely/digital-access-to-primary-care).

## Abbreviations

CI: Confidence interval
COVID-19: Coronavirus disease
HER: Electronic health record
GP: General practitioner
IMD: Index of Multiple Deprivation
LSOA: Lower Super Output Area
NHS: National Health Service
OR: Odds ratio
PR: Probability
SES: Socioeconomic status
SNOMED: Systematised Nomenclature of Medicine
TPP: The Phoenix Partnership (TPP) SystmOne software
UK: United Kingdom
WHO: World Health Organization
