# Supplementary material for: Disparities in the use of remote general practice consultations: learning from the COVID-19 pandemic, an analysis of 19 million electronic health records using OpenSAFELY
Source: BMC Med. 2025 Dec 2;23:673. doi: 10.1186/s12916-025-04469-1 (PMC12673744; doi:10.1186/s12916-025-04469-1)
Supplement: Supplementary file 1 — Additional file 1: Figures S1–S4. Fig. S1 Monthly proportion of remote consultations by age group. Fig. S2 Monthly proportion of remote consultations by gender. Fig. S3 Monthly proportion of remote consultations by ethnicity. Fig. S4 Monthly proportion of remote consultations by area deprivation quintiles. Area deprivation measured by Index of Multiple Deprivation. Tables S1–S6. Table S1 Total consultations March 2019 to March 2022 by age group and population size by age group. Table S2 Probability of a consultation being remote by age group and period. Table S3 Probability of a consultation being remote by gender and period. Table S4 Probability of a consultation being remote by area deprivation quintile and period. Table S5 Probability of a consultation being remote by ethnic group and period. Table S6 Binomial regression of the proportion of remote consultations including interaction with period [file 12916_2025_4469_MOESM1_ESM.docx]

SUPPLEMENTARY MATERIALS (including figures, tables and additional texts explaining the main document).

Trends in proportion of remote consultations 2019-2022: by age-group, gender, ethnicity and deprivation


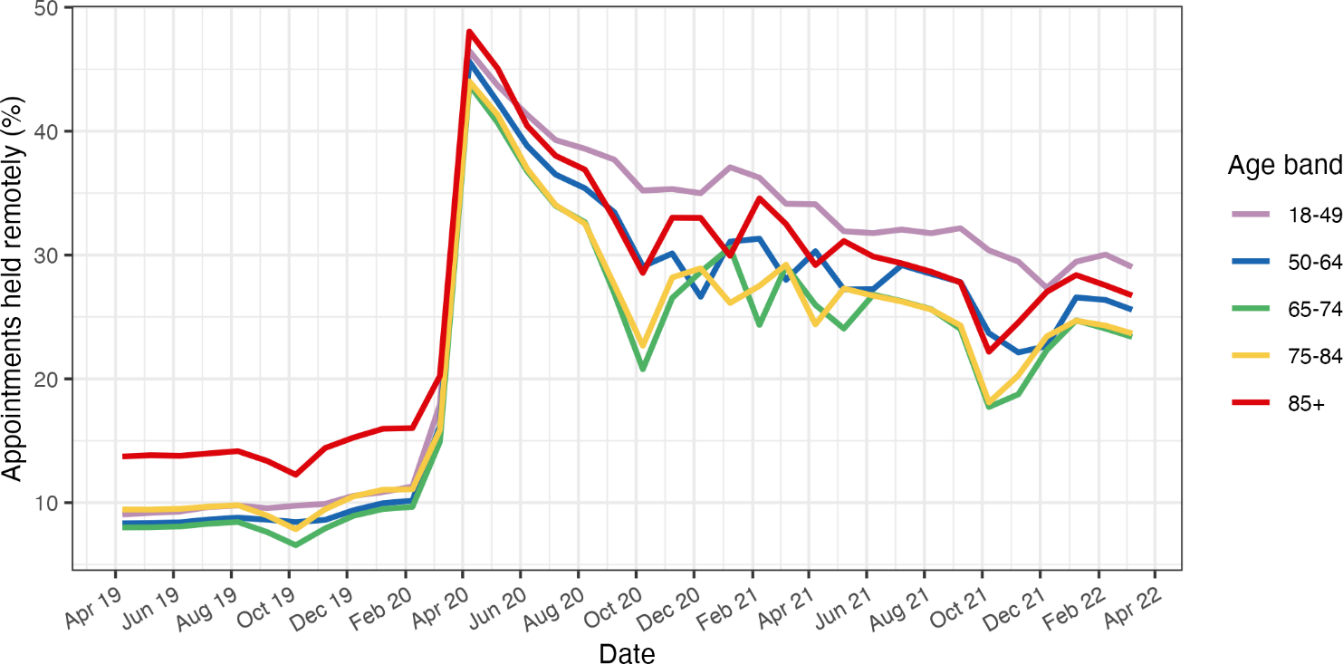


Figure SI: Monthly proportion of remote consultations by age-group (23 March 2019 to 22 March 2022).


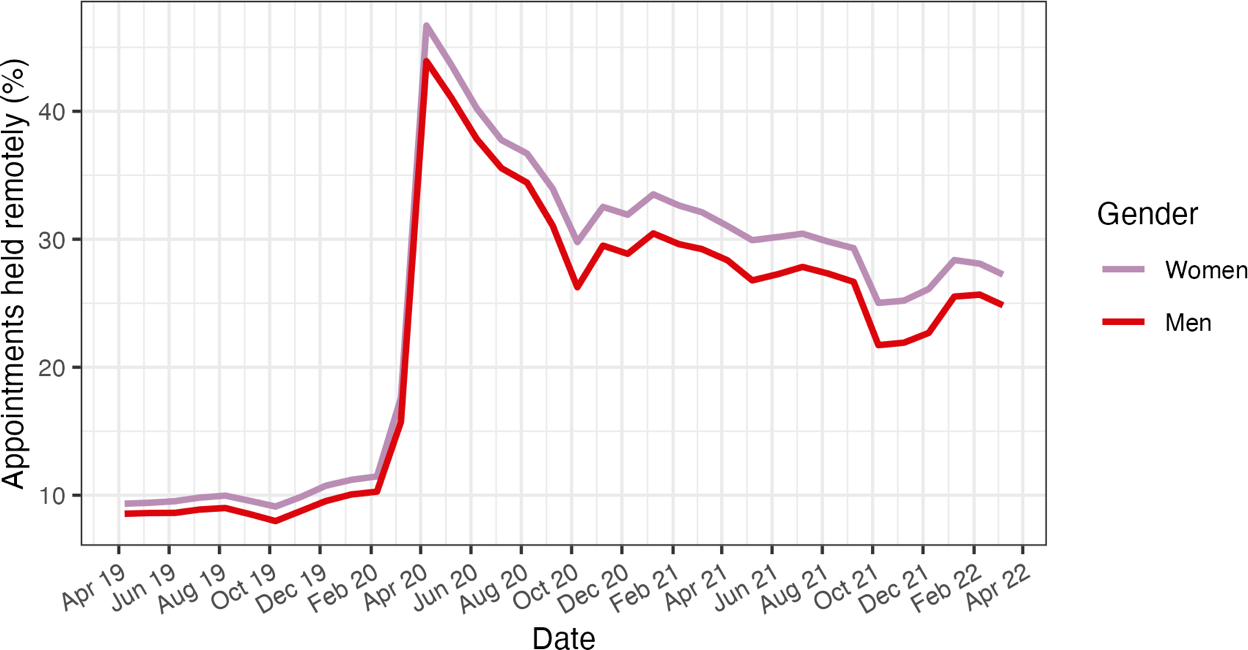


Figure S2: Monthly proportion of remote consultations by gender (23 March 2019 to 22 March 2022)


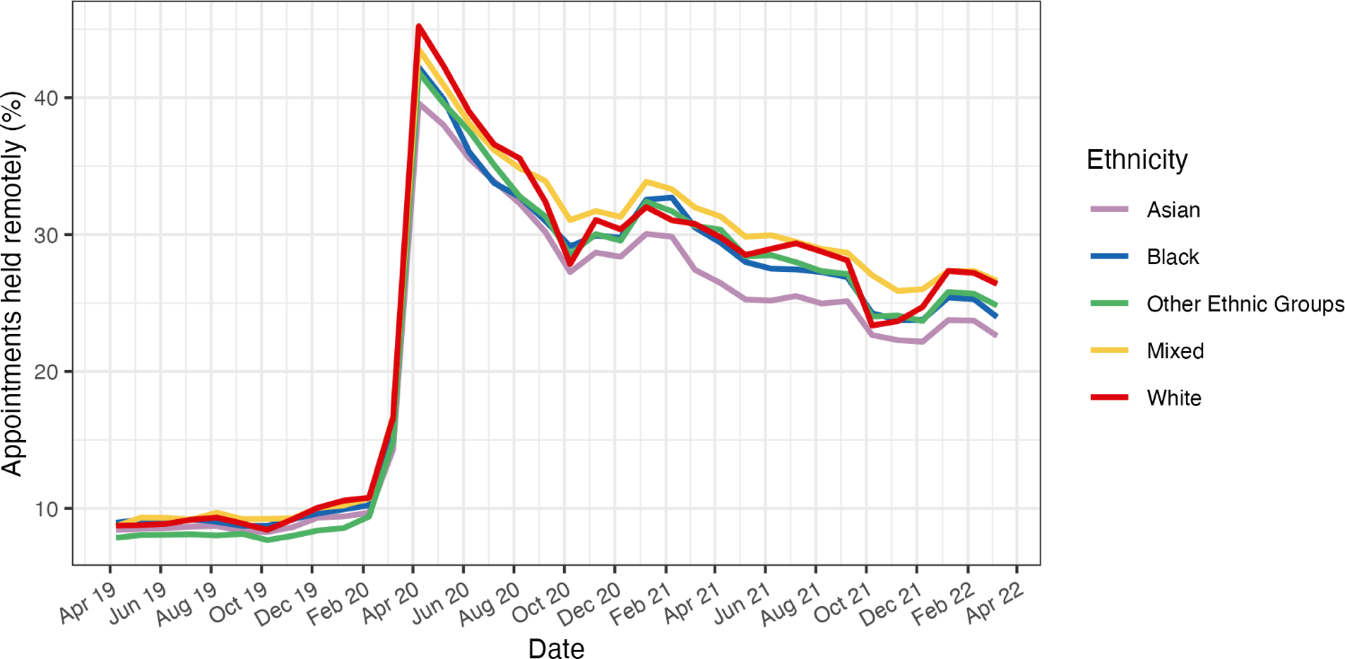


Figure S3: Monthly proportion of remote consultations by ethnicity (23 March 2019 to 22 March 2022).


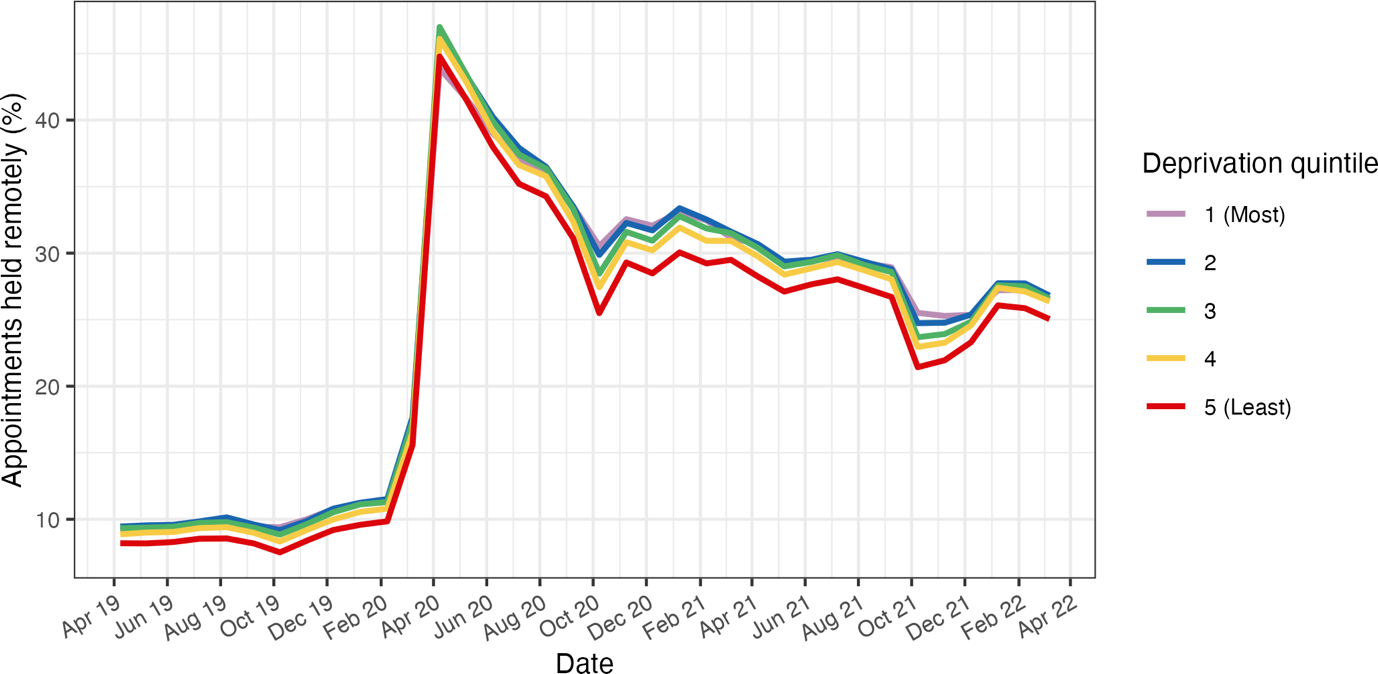


Figure S4: Monthly proportion of remote consultations by area deprivation quintiles. Area deprivation measured by Index of Multiple Deprivation (23 March 2019 to 22 March 2022).

Table S1 shows the total consultations between March 2019 to March 2022 by age-group (1000s) and population size by age-group (2021 Census).

| Table I: Total consultations March 2019 to March 2022 by age-group (1000s) and population size by age-group (2021 Census) | | | | |
| --- | --- | --- | --- | --- |
|  | Total Consultations March 2019-March 2022 | | 2021 Census Population data | |
|  | Consultations (1000s) | Percentage of consultations by age-group | Population by age-group | Percentage in age-group |
| 18-49 | 115,407 | 37.3% | 24,533,915 | 53.7% |
| 50-64 | 75,740 | 24.5% | 11,608,075 | 25.4% |
| 65-74 | 53,598 | 17.3% | 5,923,115 | 13.0% |
| 75-84 | 44,368 | 14.3% | 2,171,788 | 4.7% |
| 85+ | 20,289 | 6.6% | 1,454,740 | 3.2% |
| Total | 309,402 |  | 45,691,633 |  |

Probabilities and Odds ratio of remote consultations (Tables S2 to S6).

| Table S2: Probability of a consultation being remote by age group and period. | | | |
| --- | --- | --- | --- |
| **Period** | **Age** | **Estimate** | **95% CI** |
| 23 Mar 2019 – 22 Mar 2020 | 18-49 | 0.0960 | [0.0958-0.0963] |
| 23 Mar 2019 – 22 Mar 2020 | 50-64 | 0.0864 | [0.0862-0.0867] |
| 23 Mar 2019 – 22 Mar 2020 | 65-74 | 0.0798 | [0.0796-0.0800] |
| 23 Mar 2019 – 22 Mar 2020 | 75-84 | 0.0918 | [0.0916-0.0921] |
| 23 Mar 2019 – 22 Mar 2020 | 85+ | 0.1329 | [0.1324-0.1333] |
| 23 Mar 2020 – 22 Mar 2021 | 18-49 | 0.3501 | [0.3498-0.3505] |
| 23 Mar 2020 – 22 Mar 2021 | 50-64 | 0.3055 | [0.3051-0.3059] |
| 23 Mar 2020 – 22 Mar 2021 | 65-74 | 0.2746 | [0.2742-0.2750] |
| 23 Mar 2020 – 22 Mar 2021 | 75-84 | 0.2790 | [0.2786-0.2794] |
| 23 Mar 2020 – 22 Mar 2021 | 85+ | 0.3205 | [0.3200-0.3210] |
| 23 Mar 2021 – 22 Mar 2022 | 18-49 | 0.2824 | [0.2821-0.2827] |
| 23 Mar 2021 – 22 Mar 2022 | 50-64 | 0.2408 | [0.2405-0.2411] |
| 23 Mar 2021 – 22 Mar 2022 | 65-74 | 0.2131 | [0.2127-0.2134] |
| 23 Mar 2021 – 22 Mar 2022 | 75-84 | 0.2156 | [0.2153-0.2159] |
| 23 Mar 2021 – 22 Mar 2022 | 85+ | 0.2465 | [0.2461-0.2470] |

| Table S3: Probability of a consultation being remote by gender and period | | | |
| --- | --- | --- | --- |
| **Period** | Gender | **Estimate** | **95% CI** |
| 23 Mar 2019 – 22 Mar 2020 | Women | 0.1018 | [0.1015-0.1020] |
| 23 Mar 2019 – 22 Mar 2020 | Men | 0.0930 | [0.0928-0.0933] |
| 23 Mar 2020 – 22 Mar 2021 | Women | 0.3153 | [0.3149-0.3156] |
| 23 Mar 2020 – 22 Mar 2021 | Men | 0.2966 | [0.2963-0.2970] |
| 23 Mar 2021 – 22 Mar 2022 | Women | 0.2494 | [0.2492-0.2497] |
| 23 Mar 2021 – 22 Mar 2022 | Men | 0.2299 | [0.2296-0.2302] |

| Table S4: Probability of a consultation being remote by area deprivation quintile and period. | | | |
| --- | --- | --- | --- |
| **Period** | **Deprivation quintile** | **Estimate** | **95% CI** |
| 23 Mar 2019 – 22 Mar 2020 | 1 (Most) | 0.1028 | [0.1025-0.1030] |
| 23 Mar 2019 – 22 Mar 2020 | 2 | 0.1035 | [0.1032-0.1037] |
| 23 Mar 2019 – 22 Mar 2020 | 3 | 0.1001 | [0.0999-0.1004] |
| 23 Mar 2019 – 22 Mar 2020 | 4 | 0.0944 | [0.0942-0.0947] |
| 23 Mar 2019 – 22 Mar 2020 | 5 (Least) | 0.0862 | [0.0860-0.0864] |
| 23 Mar 2020 – 22 Mar 2021 | 1 (Most) | 0.3111 | [0.3107-0.3115] |
| 23 Mar 2020 – 22 Mar 2021 | 2 | 0.3154 | [0.3151-0.3158] |
| 23 Mar 2020 – 22 Mar 2021 | 3 | 0.3107 | [0.3103-0.3111] |
| 23 Mar 2020 – 22 Mar 2021 | 4 | 0.3028 | [0.3024-0.3031] |
| 23 Mar 2020 – 22 Mar 2021 | 5 (Least) | 0.2898 | [0.2894-0.2902] |
| 23 Mar 2021 – 22 Mar 2022 | 1 (Most) | 0.2447 | [0.2444-0.2450] |
| 23 Mar 2021 – 22 Mar 2022 | 2 | 0.2456 | [0.2453-0.2459] |
| 23 Mar 2021 – 22 Mar 2022 | 3 | 0.2418 | [0.2415-0.2421] |
| 23 Mar 2021 – 22 Mar 2022 | 4 | 0.2385 | [0.2382-0.2388] |
| 23 Mar 2021 – 22 Mar 2022 | 5 (Least) | 0.2278 | [0.2275-0.2281] |

| Table S5: Probability of a consultation being remote by ethnic group and period. | | | |
| --- | --- | --- | --- |
| **Period** | **Ethnicity** | **Estimate** | **95% CI** |
| 23 Mar 2019 – 22 Mar 2020 | Asian | 0.0931 | [0.0928-0.0933] |
| 23 Mar 2019 – 22 Mar 2020 | Black | 0.0981 | [0.0977-0.0986] |
| 23 Mar 2019 – 22 Mar 2020 | Other | 0.0895 | [0.0889-0.0901] |
| 23 Mar 2019 – 22 Mar 2020 | Mixed | 0.1025 | [0.1018-0.1031] |
| 23 Mar 2019 – 22 Mar 2020 | White | 0.1038 | [0.1037-0.1039] |
| 23 Mar 2020 – 22 Mar 2021 | Asian | 0.2866 | [0.2863-0.2870] |
| 23 Mar 2020 – 22 Mar 2021 | Black | 0.3012 | [0.3005-0.3019] |
| 23 Mar 2020 – 22 Mar 2021 | Other | 0.3019 | [0.3010-0.3028] |
| 23 Mar 2020 – 22 Mar 2021 | Mixed | 0.3126 | [0.3116-0.3135] |
| 23 Mar 2020 – 22 Mar 2021 | White | 0.3275 | [0.3273-0.3276] |
| 23 Mar 2021 – 22 Mar 2022 | Asian | 0.2171 | [0.2168-0.2174] |
| 23 Mar 2021 – 22 Mar 2022 | Black | 0.2336 | [0.2330-0.2342] |
| 23 Mar 2021 – 22 Mar 2022 | Other | 0.2381 | [0.2374-0.2389] |
| 23 Mar 2021 – 22 Mar 2022 | Mixed | 0.2491 | [0.2483-0.2499] |
| 23 Mar 2021 – 22 Mar 2022 | White | 0.2605 | [0.2604-0.2606] |

| Table S6: Binomial regression of the proportion of remote consultations including interaction with period | | | |
| --- | --- | --- | --- |
|  | Regression model with interactions | | |
|  | 23 Mar 2019 – 22 Mar 2020^a^ | 23 Mar 2020 – 22 Mar 2021^b^ | 23 Mar 2021 – 22 Mar 2022^b^ |
|  | Odds OR, [95% CI] | Odds OR, [95% CI] | Odds OR, [95% CI] |
| **Age group** |  |  |  |
| 18-49 years | Ref | Ref | Ref |
| 50-64 years | 0.89 [0.88-0.89] | 0.91 [0.91-0.92] | 0.90 [0.90-0.91] |
| 65-74 years | 0.82 [0.81-0.82] | 0.86 [0.85-0.86] | 0.84 [0.84-0.84] |
| 75-84 years | 0.95 [0.94-0.95] | 0.75 [0.75-0.76] | 0.73 [0.73-0.74] |
| 85+ years | 1.44 [1.43-1.44] | 0.60 [0.60-0.61] | 0.57 [0.57-0.58] |
| **Gender** |  |  |  |
| Women | Ref | Ref | Ref |
| Men | 0.90 [0.90-0.91] | 0.90 [0.90-0.91] | 0.99 [0.99-0.99] |
| **Deprivation** |  |  |  |
| 1 (Most) | Ref | Ref | Ref |
| 2 | 1.00 [1.00-1.01] | 1.01 [1.00-1.01] | 0.99 [0.99-1.00] |
| 3 | 0.97 [0.96-0.97] | 1.02 [1.02-1.03] | 1.01 [1.01-1.02] |
| 4 | 0.91 [0.90-0.91] | 1.05 [1.05-1.06] | 1.06 [1.05-1.06] |
| 5 (Least) | 0.82 [0.82-0.83] | 1.09 [1.09-1.10] | 1.10 [1.10-1.11] |
| **Ethnicity** |  |  |  |
| White | Ref | Ref | Ref |
| Asian | 0.88 [0.88-0.89] | 0.93[0.92-0.93] | 0.88[0.88-0.89] |
| Black or Black British | 0.94 [0.93-0.94] | 0.94[0.93-0.94] | 0.92[0.91-0.92] |
| Other Ethnic Groups | 0.84 [0.84-0.85] | 1.04[1.03-1.05] | 1.04[1.03-1.05] |
| Mixed | 0.98 [0.97-0.99] | 0.94[0.93-0.95] | 0.95[0.94-0.96] |
| The regression model:  Proportion of remote consultation (Y) = Age+Gender+Ethnicity+IMD+Period+Age⨯Period+Gender⨯Period+Ethnicity⨯Period+IMD⨯Period,  where Period indicates 23 Mar 2019 – 22 Mar 2020 (=0), 23 Mar 2020 – 22 Mar 2021 (=1), and 23 Mar 2021 – 22 Mar 2022.   1. Main effect 2. Interaction effect | | | |

Code list:

Codes were validated against commonly used NHS digital codes for GP appointments and published on [Opencodelist](https://www.opencodelists.org/codelist/user/KatieDavies_1234/virtual-consultation/17b2bb3d/#full-list).

| Snomed codes | Definition | |
| --- | --- | --- |
| 11797002 | Telephone call by physician to patient or for consultation | |
| 76740001 | Psychiatric telephone consultation or therapy with patient | |
| 142767001 | Test result to patient by telephone | |
| 151360008 | Patient given telephone advice out of hours | |
| 153505005 | Telephone encounter | |
| 153508007 | Encounter by computer link | |
| 165357005 | Test result to patient by telephone | |
| 183089000 | Patient given telephone advice out of hours | |
| 185317003 | Telephone encounter | |
| 185320006 | Encounter by computer link | |
| 313184003 | Patient given telephone advice out of hours | |
| 386472008 | Telephone consultation | |
| 386473003 | Telephone follow-up |  |
| 389918006 | Patient given telephone advice during surgery hours | |
| 390652002 | Patient given telephone advice during surgery hours | |
| 390942004 | Patient given telephone advice during surgery hours | |
| 401267002 | Telephone triage encounter | |
| 445450000 | Encounter by short message service text messaging | |
| 448337001 | Telemedicine consultation with patient | |
| 719410009 | Consultation via video conference | |
| 763184009 | Telepractice consultation | |
| 24671000000101 | Telephone call to a patient | |
| 24681000000104 | Telephone consultation | |
| 35491000000103 | Telephone call to a patient | |
| 35501000000109 | Telephone call to a patient | |
| 35531000000103 | Telephone consultation | |
| 35541000000107 | Telephone consultation | |
| 149971000000103 | Encounter using general practice online consultation system | |
| 279961000000108 | Nurse telephone triage | |
| 325871000000103 | Remote consultation encounter type | |
| 325881000000101 | Remote verbal consultation encounter type | |
| 325891000000104 | Consultation by telephone encounter type | |
| 325901000000103 | Remote non-verbal consultation encounter type | |
| 325911000000101 | Consultation via multimedia encounter type | |
| 325921000000107 | Consultation via video conference encounter type | |
| 325951000000102 | Remote assessment encounter type | |
| 325961000000104 | Remote verbal assessment encounter type | |
| 325971000000106 | Assessment by telephone encounter type | |
| 325981000000108 | Remote non-verbal assessment encounter type | |
| 325991000000105 | Assessment via multimedia encounter type | |
| 326001000000109 | Assessment via video conference encounter type | |
| 520141000000108 | Telephone consultation for suspected influenza A virus subtype H1N1 | |
| 520151000000106 | Telephone consultation for suspected influenza A virus subtype H1N1 | |
| 520171000000102 | Advice given about influenza A virus subtype H1N1 by telephone | |
| 520181000000100 | Advice given about influenza A virus subtype H1N1 by telephone | |
| 557761000005104 | E-mail consultation |  |
| 773531000000103 | Telemedicine consultation | |
| 854891000000104 | Telehealth encounter type | |
| 885661000000103 | Able to participate in telemedicine consultation | |
| 978871000000104 | Consultation via multimedia | |
| 978881000000102 | Consultation via video conference | |
| 1068881000000101 | eConsultation via online application | |
| 1083031000000104 | Telepractice consultation | |
| 1240451000000106 | Telephone consultation for suspected severe acute respiratory syndrome coronavirus 2 | |
| 1240731000000107 | Advice given about severe acute respiratory syndrome coronavirus 2 by telephone | |
| 1323941000000101 | Group consultation via video conference | |
| 1479971000168109 | Email encounter |  |

Information Governance:

NHS England is the data controller for OpenSAFELY-TPP; [TPP is the data processor]; all study authors using OpenSAFELY have the approval of NHS England [1]. This implementation of OpenSAFELY is hosted within the [TPP environment which is] accredited to the ISO 27001 information security standard and is NHS IG Toolkit compliant [2]; Patient data has been pseudonymised for analysis and linkage using industry standard cryptographic hashing techniques; all pseudonymised datasets transmitted for linkage onto OpenSAFELY are encrypted; access to the platform is via a virtual private network (VPN) connection, restricted to a small group of researchers; the researchers hold contracts with NHS England and only access the platform to initiate database queries and statistical models; all database activity is logged; only aggregate statistical outputs leave the platform environment following best practice for anonymisation of results such as statistical disclosure control for low cell counts [3]. The OpenSAFELY research platform adheres to the obligations of the UK General Data Protection Regulation (GDPR) and the Data Protection Act 2018. In March 2020, the Secretary of State for Health and Social Care used powers under the UK Health Service (Control of Patient Information) Regulations 2002 (COPI) to require organisations to process confidential patient information for the purposes of protecting public health, providing healthcare services to the public and monitoring and managing the COVID-19 outbreak and incidents of exposure; this sets aside the requirement for patient consent [4]. This was extended in November 2022 for the NHS England OpenSAFELY COVID-19 research platform [5]. In some cases of data sharing, the common law duty of confidence is met using, for example, patient consent or support from the Health Research Authority Confidentiality Advisory Group [6]. Taken together, these provide the legal bases to link patient datasets on the OpenSAFELY platform. GP practices, from which the primary care data are obtained, are required to share relevant health information to support the public health response to the pandemic and have been informed of the OpenSAFELY analytics platform.

This study was submitted to the Health Research Authority, Research Ethics Service and approved by the West of Scotland Research Ethics Service [REC reference: 23/WS/0032] and all study authors using OpenSAFELY have the approval of NHS England. OpenSAFELY is hosted within the TPP environment which is accredited to the ISO 27001 information security standard and is NHS Information Governance Toolkit compliant. Patient data have been pseudonymised for analysis and linkage using industry standard cryptographic hashing techniques. All pseudonymised datasets transmitted for linkage onto OpenSAFELY are encrypted, and access to the NHS England OpenSAFELY COVID-19 service is via a virtual private network (VPN) connection. The researchers conducting analysis hold contracts with NHS England and only access the platform to initiate database queries and statistical models. All database activity is logged, and only aggregate statistical outputs leave the platform environment following best practice for anonymisation of results such as statistical disclosure control for low cell counts.

Data management:

All data were linked, stored, and analysed securely using the OpenSAFELY platform. Data include pseudonymised data such as clinically coded diagnoses, medications, and physiological parameters. All code is shared openly for review and re-use under Massachusetts Institute of Technology (MIT) open license (https://github.com/opensafely/digital-access-to-primary-care). Data management was performed using Python 3.8, with analysis carried out using R version 4.3.1. Statistical codes are written on a local machine and pushed to the OpenSAFELY workflow via GitHub to be tested using dummy data for operational errors. Once checked the codes can be run against the OpenSAFELY-TPP database. Live information about projects and analysis running on the jobsite can be viewed publicly (https://jobs.opensafely.org/digital-access-to-primary-care-for-older-people-during-covid/).

1. NHS Digital. The NHS England OpenSAFELY COVID-19 service - privacy notice. NHS Digital (Now NHS England). https://digital.nhs.uk/coronavirus/coronavirus-covid-19-response-information-governance-hub/the-nhs-england-opensafely-covid-19-service-privacy-notice (accessed 4 July 2023).

2. NHS Digital. Data Security and Protection Toolkit. 2020. <https://digital.nhs.uk/data-and-information/looking-after-information/data-security-and-information-governance/data-security-and-protection-toolkit> (accessed 12 December 2024)

3. NHS Digital. ISB1523: Anonymisation Standard for Publishing Health and Social Care Data. NHS Digital (Now NHS England). <https://digital.nhs.uk/data-and-information/information-standards/information-standards-and-data-collections-including-extractions/publications-and-notifications/standards-and-collections/isb1523-anonymisation-standard-for-publishing-health-and-social-care-data>  (accessed 4 July 2023)

4. Secretary of State for Health and Social Care - UK Government. Coronavirus (COVID-19): notice under regulation 3(4) of the Health Service (Control of Patient Information) Regulations 2002 – general. 2022. <https://www.gov.uk/government/publications/coronavirus-covid-19-notification-of-data-controllers-to-share-information/coronavirus-covid-19-notice-under-regulation-34-of-the-health-service-control-of-patient-information-regulations-2002-general--2> (accessed 5 July 2023)

5. Secretary of State for Health and Social Care - UK Government. Coronavirus (COVID-19): notification to organisations to share information. 2022. [https://www.gov.uk/government/publications/coronavirus-covid-19-notification-to-organisat ions-to-share-information/coronavirus-covid-19-notice-under-regulation-34-of-the-health-s ervice-control-of-patient-information-regulations-2002](https://www.gov.uk/government/publications/coronavirus-covid-19-notification-to-organisat%20ions-to-share-information/coronavirus-covid-19-notice-under-regulation-34-of-the-health-s%20ervice-control-of-patient-information-regulations-2002)

6. NHS Health Research Authority. Confidentiality Advisory Group. <https://www.hra.nhs.uk/about-us/committees-and-services/confidentiality-advisory-group/> (accessed 30 January 2023)
